# Reviewer acknowledgement 2015

**DOI:** 10.1186/s13039-015-0122-y

**Published:** 2015-03-28

**Authors:** Thomas Liehr, Henry Heng, Yuri Yurov, Aurelia Meloni-Ehrig, Ivan Iourov

**Affiliations:** Jena University Hospital, Institute of Human Genetics, Jena, Germany; Center for Molecular Medicine and Genetics, Wayne State University School of Medicine, MI, USA; National Research Center of Mental Health, Moscow, Russia; CSI Laboratories, Alpharetta, GA, USA

## Abstract

The Editors of *Molecular Cytogenetics* would like to thank all our reviewers who have contributed to the journal in volume 7 (2014).

**Lauri Aaltonen**

Finland

**Dilek Aktas**

Turkey

**Eyad Alhourani**

Syria

**Daniela Alosi**

Denmark

**Gilda Alves**

Brazil

**Bonnet-Garnier Amélie**

France

**Rouben Aroutiounian**

Armenia

**Liming Bao**

United States of America

**John Barber**

United Kingdom

**Denise Batista**

United States of America

**Elena Benavente**

Spain

**Soledad Berrios**

Chile

**Thomas Bettecken**

Germany

**David Betts**

Ireland

**Samarth Bhatt**

United States of America

**Joan Blanco**

Spain

**Maria Clara Bonaglia**

Italy

**Fatih Boyar**

United States of America

**Lukrecija Brecevic**

Croatia

**Helene Bruyere**

Canada

**The-Hung Bui**

Sweden

**Monica Bullejos**

Spain

**Joern Bullerdiek**

Germany

**J Peter Burbach**

Netherlands

**Isabel Maria Marques Carreira**

Portugal

**Arunrat Chaveerach**

Thailand

**Sau Cheung**

United States of America

**Richard Choy**

Hong Kong

**Marcelo Cioffi**

Brazil

**Carlos Cordova-Fletes**

Mexico

**Nicholas Cross**

United Kingdom

**Patricia Crotwell**

United States of America

**Marta Czako**

Hungary

**Zunyan Dai**

United States of America

**Paola Dal Cin**

United States of America

**Marc De Braekeleer**

France

**Edivaldo de Oliveira**

Brazil

**Ignacio del Castillo**

Spain

**Marie Dell'Aquila**

United States of America

**Martine Doco-Fenzy**

France

**Yassamine Doubaj**

Morocco

**Regen Drouin**

Canada

**Peter Duesberg**

United States of America

**Jean-Michel Dupont**

France

**Emel Ergul**

Turkey

**Yao-Shan Fan**

United States of America

**Jelena Filipovic**

Serbia

**Michael Francki**

Australia

**Rita Genesio**

Italy

**Mary Ann George**

Canada

**Ioannis Georgiou**

Greece

**Ad Geurts van Kessel**

Netherlands

**Roberto Giorda**

Italy

**Santhosh Girirajan**

United States of America

**John Graham**

United States of America

**Ahmed Hamid**

Denmark

**Karen Harrison**

Canada

**Nyla Heerema**

United States of America

**Simone Heidemann**

Germany

**Julia Hentschel**

Germany

**Jennelle Hodge**

United States of America

**Andreas Houben**

Germany

**Colleen Jackson-Cook**

United States of America

**Fan Jin**

China

**Vaidehi Jobanputra**

United States of America

**Steve Kargas**

United States of America

**Michael Kharas**

United States of America

**Tjitske Kleefstra**

Netherlands

**Elena Kolomietz**

Canada

**Yahui Kong**

United States of America

**Dal-Hoe Koo**

United States of America

**György Kosztolányi**

Hungary

**Dieter Kotzot**

Austria

**Alma Kuechler**

Germany

**Jürgen Kunz**

Germany

**Ingo Kurth**

Germany

**Edward Lammer**

United States of America

**Lidia Larizza**

Italy

**Sonja Levanat**

Croatia

**Marilyn Li**

United States of America

**Peining Li**

United States of America

**Caroline Mackie Ogilvie**

United Kingdom

**Kamlesh Madan**

Netherlands

**Pamela Magini**

Italy

**Marina Manvelyan**

Armenia

**Jian-Hua Mao**

United States of America

**Lucia Martelli**

Brazil

**Ludmila Matyakhina**

United States of America

**Ariadni Mavrou**

Greece

**Elizabeth McCready**

Canada

**Cristina Mecucci**

Italy

**Maria Isabel Melaragno**

Brazil

**Clemens Mellink**

Netherlands

**Joana Melo**

Portugal

**Martina Merkas**

Croatia

**Konstantin Miller**

Germany

**Anna-Maja Molin**

Sweden

**Marta Molnar-Lang**

Hungary

**Kristin Mrasek**

Germany

**Elisabeth Nacheva**

United Kingdom

**Abdelhafid Natiq**

Morocco

**Yi Ning**

United States of America

**JunQi Niu**

China

**Beata Nowakowska**

Belgium

**Maria Oliver-Bonet**

Spain

**Moneeb Othman**

Yemen

**George Patrinos**

Greece

**Greg Peters**

Australia

**Michael Petersen**

Denmark

**Ruthann Pfau**

United States of America

**Qingwei Qi**

China

**Zhongxia Qi**

United States of America

**Petr Ráb**

Czech Republic

**Gopakumar Radhakrishnan**

India

**Aleksandar Rajkovic**

United States of America

**Terje Raudsepp**

United States of America

**Pierre Ray**

France

**Horacio Rivera**

Mexico

**Laura Rodriguez**

Spain

**Marta Rodriguez de Alba**

Spain

**Caroline Rooryck-Thambo**

France

**Nikolai Rubtsov**

Russian Federation

**Juliana Sa**

United States of America

**Antonio Sanchez**

Spain

**Heather Sanders**

United States of America

**Albert Schinzel**

Switzerland

**Claire Schwab**

United Kingdom

**Marco Seri**

Italy

**Frenny Sheth**

India

**Harsh Sheth**

United Kingdom

**Photini Sinnis**

United States of America

**Carolina Sismani**

Cyprus

**Dominique Smeets**

Netherlands

**Young Bae Sohn**

South Korea

**Jeremy Squire**

Brazil

**Pawel Stankiewicz**

United States of America

**Joshua Stevens**

United States of America

**Michael Talkowski**

United States of America

**Jan Traeger-Synodinos**

Greece

**Vladimir Trifonov**

Russian Federation

**Mars van't Veer**

United Kingdom

**Roberta Vanni**

Italy

**Joris Vermeesch**

Belgium

**Társis Vieira**

Brazil

**Olaya Villa**

Spain

**Emanuela Volpi**

United Kingdom

**Els Voorhoeve**

Netherlands

**Svetlana Vorsanova**

Russian Federation

**Abdulsamad Wafa**

Syria

**Meaghan Wall**

Australia

**Kui Wang**

China

**Jingly Weier**

United States of America

**Heinz-Ulrich Weier**

United States of America

**Anja Weise**

Germany

**Daynna Wolff**

United States of America

**Zhengfeng Xu**

China

**Fengtang Yang**

United Kingdom

**Mingjun Zhang**

United States of America

**Ying Zou**

United States of America

